# The Current Role of Graphene-Based Nanomaterials in the Sample Preparation Arena

**DOI:** 10.3389/fchem.2020.00664

**Published:** 2020-08-11

**Authors:** Edvaldo Vasconcelos Soares Maciel, Karen Mejía-Carmona, Marcela Jordan-Sinisterra, Luis Felipe da Silva, Deyber Arley Vargas Medina, Fernando Mauro Lanças

**Affiliations:** Laboratory of Chromatography (CROMA), São Carlos Institute of Chemistry (IQSC), University of São Paulo, São Carlos, Brazil

**Keywords:** graphene, graphene oxide, cyclodextrin, molecularly-imprinted polymer, magnetic, ionic liquid, sample preparation

## Abstract

Since its discovery in 2004 by Novoselov et al., graphene has attracted increasing attention in the scientific community due to its excellent physical and chemical properties, such as thermal/mechanical resistance, electronic stability, high Young's modulus, and fast mobility of charged atoms. In addition, other remarkable characteristics support its use in analytical chemistry, especially as sorbent. For these reasons, graphene-based materials (GBMs) have been used as a promising material in sample preparation. Graphene and graphene oxide, owing to their excellent physical and chemical properties as a large surface area, good mechanical strength, thermal stability, and delocalized π-electrons, are ideal sorbents, especially for molecules containing aromatic rings. They have been used in several sample preparation techniques such as solid-phase extraction (SPE), stir bar sorptive extraction (SBSE), magnetic solid-phase extraction (MSPE), as well as in miniaturized modes as solid-phase microextraction (SPME) in their different configurations. However, the reduced size and weight of graphene sheets can limit their use since they commonly aggregate to each other, causing clogging in high-pressure extractive devices. One way to overcome it and other drawbacks consists of covalently attaching the graphene sheets to support materials (e.g., silica, polymers, and magnetically modified supports). Also, graphene-based materials can be further chemically modified to favor some interactions with specific analytes, resulting in more efficient hybrid sorbents with higher selectivity for specific chemical classes. As a result of this wide variety of graphene-based sorbents, several studies have shown the current potential of applying GBMs in different fields such as food, biological, pharmaceutical, and environmental applications. Within such a context, this review will focus on the last five years of achievements in graphene-based materials for sample preparation techniques highlighting their synthesis, chemical structure, and potential application for the extraction of target analytes in different complex matrices.

## Introduction

Over the last decades, nanotechnology has become a promising tool in relevant scientific fields, allowing humanity to reach top levels of quality in several areas such as engineering, chemistry, medicine, and sports, among others (Lin et al., 2019). One of the most significant achievements in this context was the confirmation of the existence of a single-layered graphene sheet obtained through mechanical exfoliation by Novoselov et al. in 2004 at Manchester University (Novoselov, 2004; Novoselov et al., 2005). The history of graphene (G) starts ~70 years ago when Landau and Peierls stated that strictly 2D crystals were thermodynamically unstable with a slight likelihood even to exist (Peierls, 1935; Landau, 1937). At that time, the scientists thought that the melting point of a thin-film of atoms decreased proportionally to its thickness leading to structure decomposition or segregation (Peierls, 1935; Landau, 1937). Therefore, the atomic monolayers' existence would only be possible to exist as an epitaxially-grown part of 3D complex structures. Nonetheless, this theory was confronted by experimental observations reported in 2004, which preceded the discovery of more than one type of 2D atomic monolayers, highlighting graphene as the most important of them (Geim and Novoselov, 2007). For this reason, graphene theoretically represents a new class of materials possessing a one-atom thickness that, due to its intrinsic properties, are creating new possibilities of practical applications and becoming a hot topic in science.

**Figure 1:**
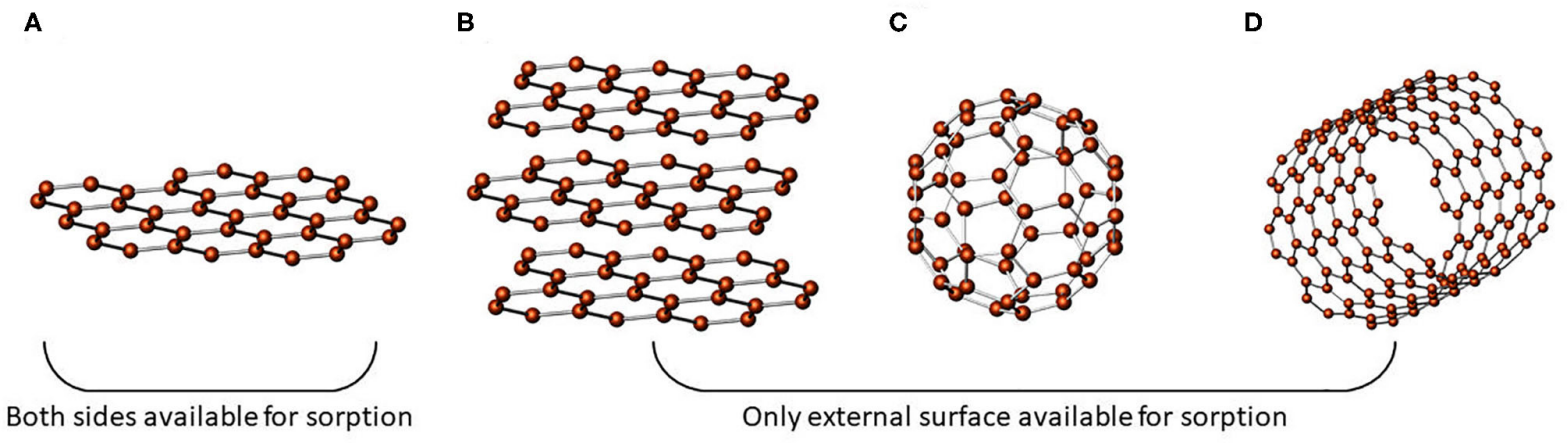
Illustrative drawing of the primary carbon allotropes: **(A)** graphene; **(B)** graphite; **(C)** fullerene; **(D)** carbon nanotubes. Adapted from Maciel et al. (2019), copyright 2019, with permission from Elsevier.

Graphene can be defined as a carbon allotrope composed by a structure containing sp^2^ hybridized atoms obeying a honeycomb pattern, which is the core for other widely-known allotropic forms (Grajek et al., 2019). In other words, graphene can be stacked to form graphite, rolled to form a carbon nanotube, or even wrapped to become a fullerene, as shown in Figure 1. In general, it is considered a wonder material due to its nanosheet structure, which has strong σ-orbitals in the 2D plane, ensuring its stiffness. At the same time, the un-hybridized π-orbitals are hinged outwards, superimposing one by one to form the long-range delocalized π electron system, responsible for its outstanding optical, and electrical properties (Grajek et al., 2019). In short, unmodified graphene sheets have a large theoretical surface area (ca. 2630 m^2^ g^−1^) distributed along with the thinnest structure of the negligible mass, but high Young's modulus, as already discovered (Fumes et al., 2015). Likewise, its charge carriers have high mobility, possibly traveling micrometers without scattering, becoming an ideal material for producing electronic devices (Hou et al., 2019). Moreover, the excellent thermal and electrical conductivity (~3,000 W mK^−1^ and 104 Ω^−1^ cm^−1^, respectively), transparency, and impermeability to gases must be underscored (Geim, 2009).

Although these top qualities suggest that graphene would be an ideal material with several different potential applications, the manufacturing (especially in industrial-scale) still represents a hurdle to its broad implementation. This occurs because the most utilized manufacturing approach is based on the graphite top-down mechanical exfoliation process by adhesive tape (Allen et al., 2010). Generally, this production method is laborious, non-reproducible, and highly dependent on human handling. It has attracted the chemists' interest in developing scalable alternative routes to produce significant amounts of high-quality graphene nanosheets. Including on this are the chemical exfoliation through liquid solutions, the bottom-up method to produce from organic precursors, among others (Allen et al., 2010; Vadivel et al., 2017). It must be noted that each of these alternative production methods has its proper characteristics. The chemical exfoliation provides interesting results regarding the quality of graphene nanosheets or even due to the existence of intermediary graphene-based compounds similarly attractive for such purposes, such as the graphite oxide or graphene oxide, for instance.

Even with the graphene existence being confirmed since 2004, its first application in sample preparation was only published in 2011 (Luo et al., 2011a; Zhang and Lee, 2011). The interest of analytical chemists on it and its derivatives is mostly due to the increasing demand for high-performance and selective materials to extract contaminants present in complex matrices containing a large number of interferents. The present outlook of our environment urges researchers to seek technological advances on sample preparation and analytical techniques to tackle even better the increasing use of chemicals by humans in many areas of life: agriculture, health-treatments, and abusive-drugs, among others. Within such a context, the necessity in performing a sample preparation step before the analytical techniques is mandatory due to the complexity related to the mostly analyzed matrices (biological fluids, food, plants, wastewaters, soil, and others). This step is crucial in the analytical workflow responsible for eliminating matrix interferents, isolating, and pre-concentrating target analytes. For these reasons, several different sorption-based sample preparation techniques (e.g., microextraction by packed sorbent [MEPS], stir bar sorptive extraction [SBSE], magnetic solid-phase extraction [MSPE], among others) have been proposed. They are mostly derived from conventional SPE and its main miniaturized mode SPME (Fumes et al., 2015). Generally, they are performed by the employment of an extractive phase, usually named as sorbent. Ideally, this sorbent must present some essential characteristics such as good selectivity to the target compounds, high extraction capability, and even is desirable as a chemical inertia for those interferents present in the analyzed matrix (Toffoli et al., 2018).

Then, GBMs emerged as promising sorbents to be used in the extraction techniques, due to its chemical structure and properties, which favors the extraction performance by effectively removing the target analytes from complex matrices. Considering all the advantages herein presented, some of them are more interesting from the analytical chemistry standpoint. For example, the flat graphene structure allows potential target analytes to interact on both sides of it, which is advantageous for sorption-based sample preparation techniques. In the case of other carbon allotropes (e.g., carbon nanotubes, graphite, and fullerene), only the external surface is available for such interaction, which potentially diminishes extraction performance due to this steric hindrance associated. Additionally, the delocalized π-electron system favors electrostatic interaction between the graphene and molecules that possess aromatic rings in its structure.

For this reason, prevalent contaminants such as pesticides, preservatives, pharmaceuticals, and veterinary drugs can be remediated from the environment by using GBMs (Toffoli et al., 2018). Contrariwise, when the potential contaminant does not have aromatic rings, a functional intermediary produced from the chemical exfoliation, namely graphene oxide (GO), can be used instead of graphene. This is owing to its chemical structure that differs from G by the presence of oxygenated groups (e.g., hydroxyl, carbonyl, alkoxy) outside of its 2D-plane, possibly favoring interactions with polar active-sites in other molecules (Smith et al., 2019). Nonetheless, from an operational point of view, the fact that graphene is an ultra-light material makes difficult its deposition by, for example, centrifugation. In this way, some chemical modification or functionalization can be performed to overcome such drawbacks. Nowadays, other carbon-based compounds that possess one-atom planar structures are beginning to spur around, mainly due to the attention given to the scientific community's graphene in the last years. Including in this group are graphyne, graphdiyne, graphone, and graphane, all considered as graphene-derivative compounds (Peng et al., 2014). As an example, graphyne and graphdiyne are 2D-flat allotropic forms of graphene possessing the same honeycomb pattern, which suggests them as suitable for similar applications as its precursor (graphene).

Conversely, graphone and graphene emerged as hydrogenated graphene-derivative compounds susceptible to chemical modifications onto its surfaces. Despite these compounds already discussed in the literature, their synthesis remains a complicated process; for this reason, they have not yet been applied in sample preparation. However, considering the significant advances in graphene-based technologies since its discovery, these other allotropic forms might gain more attention from scientists throughout the years.

Following the background regarding the emerging of graphene, this review aims to present the state-of-art about this “wonder material” from a sample preparation viewpoint. In this way, several aspects such as synthesis and functionalization process, the main derivative classes, and its most suitable applications are divided among the next sections. In short, our primary goal was to present a review mostly covering the last 5 years' achievements of the still-evolving field of graphene-based materials in sample preparation and discuss the future trends and potential challenges that chemists should face in the years to come.

## Graphene and Graphene Oxide

As mentioned, graphene (G) is a 2D monolayer of carbon atoms covalently bonded in a honeycomb pattern, displaying a flat sheet conformation (Solís-Fernández et al., 2017). Graphene and related materials are part of the graphene-based materials (GBMs), which comprises graphene (G) nanosheets (in mono, few, and multi-layers), graphene oxide (GO), and reduced graphene oxide (rGO) (De Marchi et al., 2018). A considerable variety of research articles about different synthesis methods, properties, and applications of GBMs are available (Papageorgiou et al., 2017; Lim et al., 2018; Liu and Zhou, 2019). Two different approaches can be used to obtain graphene: (i) the top-down, in which nanostructures are produced from larger dimensions, and (ii) the bottom-up, starting from atoms or small molecules to produce materials of larger dimensions.

In the top-down approach, graphene is prepared from graphite, by mechanical or chemical exfoliation, or chemical synthesis, separating the graphene thin layers parallelly stacked in the graphite and held together by weak van der Waals forces. Mechanical exfoliation is one of the simplest methods in which a simple direct contact with an adhesive tape (polymer) can take off the graphene layers from the surface of a graphite piece. One of the advantages of mechanical exfoliation is the possibility of different pile-up layers of other 2D materials with several graphene heterostructures (Solís-Fernández et al., 2017). However, it has only been implemented on a small scale and is highly susceptible to contamination. In the same way, organic solvents can be used to separate the graphite layers. Other exfoliation methods include the use of electric fields, sonication, and transfer printing technique (Lim et al., 2018). Several exfoliation methods, including different substrates, thermal released tape, and thermal approaches, have been proposed to improve the quality, size, and homogeneity of graphene (Solís-Fernández et al., 2017).

**Figure 2:**
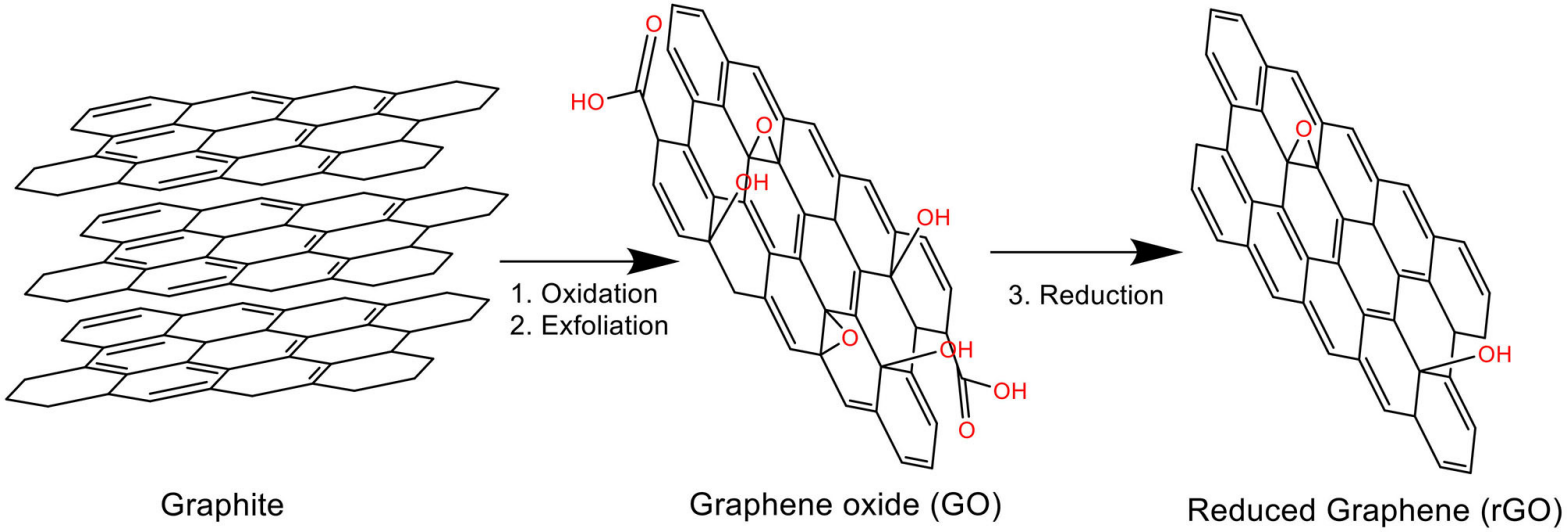
Graphene chemical reduction pathway.

Chemical reduction of graphene oxide (GO) is the most popular method to obtain graphene. As shown in Figure 2, GO can be obtained by the oxidation of graphite powder and then exfoliated further to obtain single GO layers, which is subsequently chemically reduced to obtain rGO. Although the final product obtained from this pathway is rGO, its properties are very similar to graphene but are structurally different (Dreyer et al., 2010; Singh et al., 2016). Chemical reduction of GO firstly involves exfoliation in water assisted by ultrasonication, followed by reduction of the oxygenated groups, hence precipitating the rGO from the solution due to its hydrophobicity (Singh et al., 2016). Among the wide variety of chemical reduction agents that can be employed, hydrazine is the most often used because of its high reductive efficiency, even though it is highly environmental toxic. As an alternative, the use of greener reduction agents (De Silva et al., 2017) and thermally-mediated or electrochemical reduction are also employed. Although chemical reduction of GO is a popular upscaling method, it yields a final product containing several structural defects on the sheets, which lead to low-quality materials with variable sizes and edges (Dreyer et al., 2010).

Conversely, the bottom-up approaches are another alternative to synthesize high-quality graphene layers. The leading methods used include pyrolysis, chemical vapor deposition (CVD), plasma synthesis, and epitaxial growth (Lim et al., 2018). Graphene sheets are prepared from small building blocks and assembled with dedicated precision, usually employing molecular modeling to build the layers. Among the bottom-up methods, CVD uses high temperatures for the decomposition of hydrocarbons, which are deposited on metal substrates, thus forming thin sheets of graphene (Papageorgiou et al., 2017). The main advantage of bottom-up methods is the production of high-quality graphene sheets. Nevertheless, these methods are not used for large-scale production.

Graphene oxide (GO) is like a graphene sheet functionalized on both sides with several oxygenated functions such as hydroxyl, carboxyl, and epoxy. These functions impose a hydrophilic character to GO; as a consequence, the interaction between layers is weaker compared to graphene, making GO an easily exfoliated material. GO structure depends principally on the purification methods (Singh et al., 2016). Compared to graphene, the GO structure is still ambiguous; thus, several structural models have been proposed to date (Dreyer et al., 2010; Sun, 2019). The graphene oxide can be obtained by the popular Hummer's method (Hummers and Offeman, 1958), which, until the date, has been subjected to multiple modifications and improvements (Shamaila et al., 2016). The original method proposes the oxidation of graphite powder by KMnO_4_ and NaNO_3_ in H_2_SO_4_ (Hummers and Offeman, 1958). Differences with other modified methods are principally on the type and toxicity of the oxidant reagents, and the quality of the obtained product (Lim et al., 2018).

A fascinating characteristic raised from the physical and chemical properties of graphene materials is the possibility to perform chemical functionalizations mainly to modify its reactivity yielding a large variety of graphene-based materials (GBMs). Thus, they can be currently used in several applications (Bottari et al., 2017; Mohan et al., 2018). Covalent or non-covalent pathways can functionalize GBMs. Non-covalent functionalization involves first the rupture of the van der Waals forces that stake together with the graphene layers with subsequent formation of non-covalent binding with the substrate by π-π, π-cation, and van der Waals interactions. For that, mechanical liquid exfoliation assisted by ultrasonication is used to overcome these forces; water, organic solvents, ionic liquids, surfactants, mixtures are employed to disperse, and stabilize the graphene sheets in the exfoliation process. For example, non-covalent functionalization can be obtained by forming a stable dispersion of graphene sheets in polymers (ionic, non-ionic, and polysaccharides), water solutions, and organic solvents as polyvinyl alcohol, chitosan, and alginate. They can be employed to obtain graphene aerogels and hydrogels (Dreyer et al., 2010; Bottari et al., 2017).

On the other hand, the covalent functionalization of graphene (G) yields a low substitution degree due to their stable carbon conjugation. However, covalent functionalization can be done by taking advantage of the oxygenated reactive groups of the graphene oxide (GO) sheets. Therefore, in the same way as the chemical reduction of graphene oxide, the hydroxyl, carboxyl, and epoxy groups can be covalently replaced by other functional groups. The GO surface modification with aliphatic amines to form an amide bond is one of the most common strategies (Dreyer et al., 2010). Undoubtedly, the derivatization of graphene materials improves their electrical, thermal, and mechanical properties as well as their dispersibility (Mohan et al., 2018). Some reasons for the use of functionalized graphene materials as sorbents in sample preparation include: (i) they present improved sorption capacity and recoveries; (ii) easy attachment of graphene onto surfaces to be reusable, and preventing sorbent losses; (iii) avoid the agglomeration of the graphene sheets; and (iv) favored sorbent isolation from the sample (Wang et al., 2014; Ye and Shi, 2015; Chen X. et al., 2016; González-Sálamo et al., 2016).

Considering the increasing use of GBMs in sample preparation techniques, the most representative and used materials in this arena are discussed in the following sections.

## Anchored Graphene-Based Materials

### Alkyl and Aril Groups

**Figure 3:**
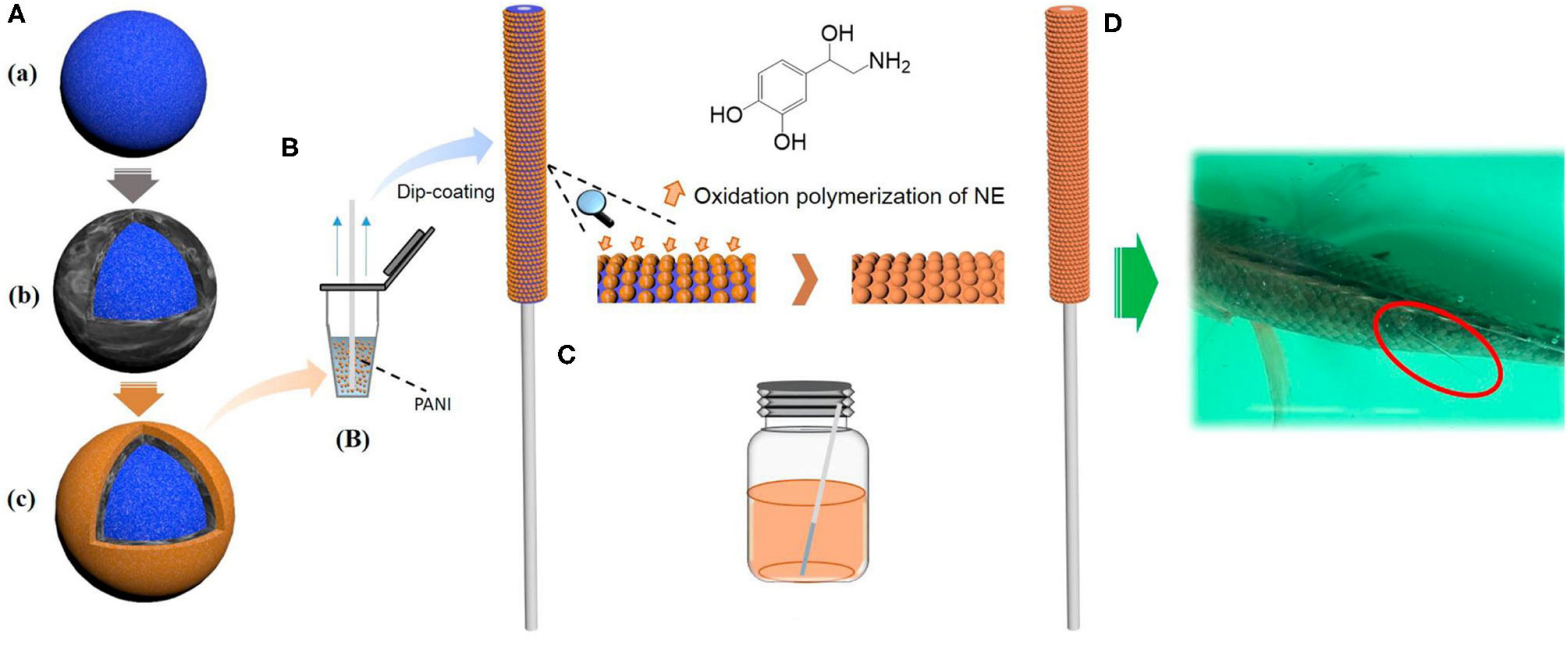
Preparation and application scheme of C18@GO@PDDA SPME fiber. **(A)** Preparation of the particles (a. C18, b. C18@GO, and c. C18@GO@PDDA). **(B)** A home-made coated PANI fiber by the dip-coating method. **(C)** Bioinspired modification by the NE oxidation polymerization. **(D)** *In vivo* sampling in fish dorsal-epaxial muscle with the fiber. Reprinted with permission from Qiu et al. (2016), copyright 2016, American Chemical Society.

Octadecylsilica particles (for short C18 or ODS) are by far the most commonly used sorbent in solid-phase extraction (SPE) and chromatographic separations. Apart from the conventional C8/C18 reversed phases, today mixed-mode polymeric sorbents are widely used in SPE because they present interactions with several compounds and better performance compared to the conventional ones, and they are also commercially available (Fontanals et al., 2020). Alkyl groups, in general, are commonly used to derivatize sorbents, including GO sheets, to modify their fundamental properties. As a consequence, C18 has also been employed to functionalize GO-based sorbents owing to the high surface area of the GO sheets. Their functionalization with octadecylsilane increases the surface load with C18 groups compared to silica particles (Liang et al., 2012; Xu et al., 2012). Subsequently, the extraction capacity is improved, and hydrophobic interactions increased. Therefore, being applied as a sorbet in reverse-phase, they show an improved extraction efficiency for the extraction of alkanes and PAHs, for instance (Xu et al., 2012). Recently, Qui et al. prepared a solid-phase microextraction (SPME) fiber with C18 particles (3.5 μm) coated with GO and poly(diallyl dimethylammonium chloride) (PDDA)–C18@GO@PDDA as shown in Figures 3A,B. Then, the surface of the fiber was modified by oxidative polymerization by polynorepinephrine (pNE) (Figure 3C), which plays the role of a bio-interface, compatible with *in-vivo* sampling (Figure 3D). The prepared SPME fibers showed higher efficiency than commercially available ones such as polydimethylsiloxane (PDMS) and polyacrylate (PA) for the monitoring of acidic drugs in fish samples. Additionally, the fiber exhibited excellent stability, sensitivity, and resistance for *in-vivo* matrices, showing potential for pharmacokinetics applications (Qiu et al., 2016). In another study, the same fiber type was successfully employed to analyze salicylic acid traces in plants *in-vivo* (Fang et al., 2018).

Although functionalization of GO occupies or replaces part of their original active sites, the sorption capacity of modified-GO materials can be lower or higher compared to GO, which mainly depends on the composite type formed and their specific interaction with the analytes. Even so, GBMs show superior sorptive properties compared to conventional sorbents (e.g., C18), which allows the use of graphene sorbents in small quantities (<100 mg) (Sitko et al., 2013). In this way, Ma et al. functionalized graphene oxide (GO) sheets with different amine-alkyl chains to obtain amine-rGO sorbents *via* solvothermal synthesis. The sorption capacity of the different alkyl-amine-rGO materials was evaluated for the extraction of catechins and caffeine. Results showed that tributylamine-rGO has the highest sorption capacity (203.7 mg g^−1^) for catechins being 11 times higher compared to GO sheets (18.7 mg g^−1^) and other rGO-amino groups (ammonia, ethylenediamine, n-butylamine, tert-butylamine, dodecyl amine, and octadecyl amine). Hence, tributylamine-rGO was employed as a sorbent in a modified QuEChERS (quick, easy, cheap, effective, rugged, and safe) method achieving a higher clean-up performance compared to traditional sorbents as PSA, C18, and graphitized carbon black (GCB), regularly used in QuERChERS (Ma et al., 2018). A similar comparison was performed by Fumes et al., which employed aminopropyl silica and PSA particles coated by graphene sheets. The extraction performance of the GO-coated particles, used as a sorbent in microextraction by packed sorbent (MEPS) method, were compared with conventional sorbents (C18, strata-X, PSA, amino silica) for the extraction of parabens in wastewater. Aminopropyl silica coated with GO (SiGO) and G (SiG) showed an improved extraction performance compared to conventional sorbents (Fumes and Lanças, 2017). Likewise, a recent work performed by the same research group showed improvements in the extraction capability of aminopropyl silica-GO particles when they are functionalized with C18 and further end-capped. The authors achieved low LODs and LOQs in a complex matrix (coffee samples) by using these particles in a packed in-tube SPME device (Mejía-Carmona and Lanças, 2020). Other interesting graphene-based applications carried out by the Lança's research group also includes tetracyclines' analysis in milk samples (Vasconcelos Soares Maciel et al., 2018) and the determination of triazines in environmental water samples (De Toffoli et al., 2018).

Several additional complex alkyl and aryl compounds have been used to functionalize GO. For example, Nurerk et al. synthesized a hybrid sorbent based on calix[4]arene-functionalized graphene oxide/polydopamine-coated cellulose acetate fiber (calix[4]arene-GO/PDA-CFs) for the extraction of aflatoxins in corn samples. Calix[4]arene is a macrocyclic molecule of four phenol units bonded by methylene bridges, which can favor the extraction of aflatoxins by H-bonding, hydrophobic, and π-π interactions. The recoveries of aflatoxins (AFs) obtained employing cellulose acetate CFs (35–41%), polydopamine coated CFs (PDA-CFs) (45–55%), calix[4]arene-GO-CFs (60–72%), GO/PDA-CFs (63–82%), and calix[4]arene-GO/PDA-CFs (86–94%) as SPE sorbents showed that together calix[4]arene and GO increased the efficiency of the sorbent phase (Nurerk et al., 2018). Recently, Zhou et al. synthesized a graphene oxide framework (GOF), a 3D nanoporous material, as coating sorbent for stir bar sorptive extraction (SBSE). Graphene oxide was covalently interconnected with a 1,4-phenylene diisocyanate (PPDI) to obtain three-dimensional GOF, which was immobilized onto the surface of stainless-steel wire (SSW) using polydopamine. The stir bar was applied successfully for the extraction of Sudan dyes in lake water and fruit juice (Zhou J. et al., 2019). Other recently published papers on alkyl and aryl modified graphene materials are shown in Table 1.

**Table 1:** Recent applications (2015–2020) of alkyl and aryl functionalized graphene-based materials in sample preparation.

**Sorbent**	**Analytes**	**Matrix**	**Sample preparation**	**Analysis**	**LOD**	**References**
Diallyl dimethyl ammonium chloride-assembled GO-coated C18 (C18@GO@PDDA)	Salicylic acid and derivates	Aloe plants (*in-vivo* sampling)	SPME fiber	HPLC-DAD	1.8–2.8 μg g^−1^	Fang et al., 2018
Aminopropyl silica coated GO- functionalized Octadecylsilane/end-capped (SiGOC18ecap)	Xanthines	Coffee	In-tube SPME	HPLC- MS/MS	0.1–0.2 μg L^−1^	Mejía-Carmona and Lanças, 2020
Graphene derivatized silica	Fluoroquinolones	Water	SPE	HPLC-FLD	2 ng L^−1^	Speltini et al., 2015
Guanidyl-functionalized GO-grafted silica (Guanidyl@GO@sil)	Herbicides	*Lycium barbarum*	SPE	HPLC-UV	0.5–2.0 μg L^−1^	Hou et al., 2018b
Polypyrrole-coated GO and C18 incorporated in chitosan cryogel (PPY/GOx/C18/CS)	Carbamate pesticides	Fruit juices	SPE	HPLC-UV	0.5–2.0 μg L^−1^	Klongklaew et al., 2018
Alkyl-NH_2_/rGO	Pesticides	Tea	Modified QuEChERS	GC-MS/MS UHPLC-MS/MS	0.33–9.26 μg kg^−1^	Ma et al., 2018
Calix[4]arene-functionalized GO/polydopamine-coated cellulose acetate fiber (calix[4]arene-GO/PDA-CFs)	Aflatoxins	Corn	SPE	HPLC-FLD	0.01–0.05 μg kg^−1^	Nurerk et al., 2018
Graphene oxide supported on aminopropyl silica (Si-GO)	Tetracyclins	Bovine milk	MEPS	HPLC-MS/MS	0.03–0.21 μg L^−1^	Vasconcelos Soares Maciel et al., 2018
Graphene oxide supported on aminopropyl silica (Si-GO)	Triazines	Water	In-tube SPME	HPLC-MS/MS	1.1–2.9 ng L^−1^	De Toffoli et al., 2018
Graphene-C18 Reinforced Hollow Fiber (G-C18-HF)	Chlorophenols	Honey	HF-LPME	HPLC-UV	0.5–1.5 μg kg^−1^	Sun et al., 2014
Poly(diallyldimethylammoniumchloride) assembled GO-coated C18 particles (C18@GO@PDDA)	Acidic pharmaceuticals	Fish (*in-vivo* sampling)	SPME fiber	HPLC-MS/MS	0.13–7.56 μg kg^−1^	Qiu et al., 2016
Graphene oxide/silica modified with nitro-substituted tris(indolyl)methane	Organic acids	Honey and nongfu spring drink	SPE	HPLC-DAD	0.5–1.0 μg L^−1^	Wang N. et al., 2016
Graphene supported on aminopropyl silica (Si-G) and primary-secondary amine (PSA) silica (PSA-G)	Parabens	Water	MEPS	UHPLC-MS/MS	0.06–0.09 μg L^−1^	Fumes and Lanças, 2017
Acrylamide-functionalized graphene	Monoamine acidic metabolites	Urine and plasma	μSPE	HPLC-UV	0.08–0.25 μg L^−1^	Yang et al., 2015
Tannic acid functionalized graphene	Beryllium	Wastewater and street dust	d-SPE	Atomic absorption	0.84 ng L^−1^	Yavuz et al., 2018
GO framework interconnected by 1,4-phenylene diisocyanate (PPDI)	Sudan dyes (G, I, II, and III)	Lake water and fruit juice	SBSE	HPLC-UV	0.15–0.3 μg L^−1^	Zhou J. et al., 2019

### Cyclodextrins

Other emergent graphene-based materials for sample preparation are those combined with cyclodextrins (CD), which are cyclic oligosaccharides formed by starch enzymatic degradation, and are linked by several α-1,4-anhydroglucopyranose. In nature, they are made up of 6, 7, or 8 glucose units and categorized as α, β, and γ-CD, respectively. CDs have cone shapes with a hydrophobic cavity and a hydrophilic external surface. This specific structure allows them to form inclusion complexes with specific molecules such as polyphenolics compounds through hydrogen bonding, hydrophobic, and Van der Waals interactions (Pinho et al., 2014; Zhu et al., 2016). Reactive OH groups can also be replaced to modify their solubility, improve inclusion ability, or induce desired properties as functionalization for immobilization on a solid support. Consequently, more than 100 CDs are commercially available, and more than 1,500 derivatives have been synthesized for different purposes (Szejtli, 2004; Gentili, 2020).

**Figure 4:**
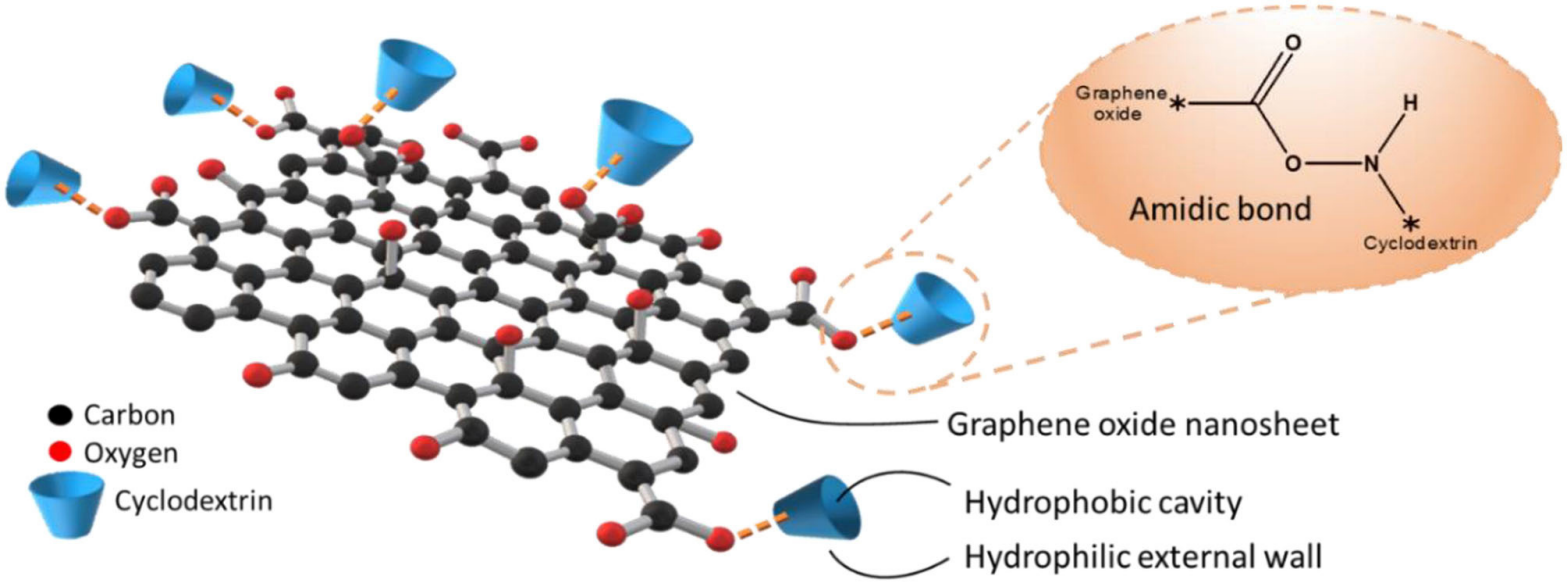
Scheme illustrating a graphene-based sorbent functionalized with cyclodextrins through peptide bonds.

The reliable recognition capacity of phenolic compounds, due to the excellent size match, becomes common to find works reporting a combination between graphene and cyclodextrins used as electrochemical detectors (Wang C. et al., 2016), electrocatalytic detector (Pham et al., 2016), and electrocatalytic material, for instance (Ran et al., 2017). Thus, there are many different strategies available in the literature to synthesize GBMs functionalized by cyclodextrins. GO functionalization with CD can be very simple, as reported by Cao et al. (2019). They prepared a suspension containing a graphene-based material and β-cyclodextrin, with it subsequently stirred under a heated water bath at a temperature of 60°C for 4 h. In this case, the resulting material was GO; if it is necessary to reduce graphene oxide to graphene, it can be done using hydrazine (Pham et al., 2016; Tan and Hu, 2017). As an example, modifications in the synthesis route can be made with (3-aminopropyl)triethoxysilane (APTES) to support an amino group to both graphene oxide nanosheets or cyclodextrins walls (Deng et al., 2017). This procedure results in an amido bonding between epoxy and –COOH groups of GO and –NH_2_ from the APTES. Figure 4 shows a scheme to exemplify the most common bond between GO and cyclodextrin, and how it is expected to be the structure of the graphene-based material functionalized by cyclodextrin.

The combination of graphene and cyclodextrins properties becomes the resulting material attractive to be employed in sample preparation techniques. An interesting application was carried out by Deng et al. The novel β-CD–GO-coated SPME fiber was prepared using a sol-gel technique and immobilizing onto a pre-functionalized stainless steel wire (Deng et al., 2017). They applied this material as a sorbent in a headspace technique (HS-SPME), aiming to extract organophosphate flame retardants in water samples to be analyzed by gas chromatography with nitrogen phosphorus detector (NPD). The method showed functional recovery (82.1–116.9%), linear range with correlation coefficients (*R*) ranging from 0.9955 to 0.9998. The LODs and LOQs for the nine analytes ranged from 1.1–60.4 to 2.7–170.5 ng L^−1^, respectively; RSD was 2.2–9.6%, and enrichment factors obtained from 22.5 to 1307.5. This high enrichment factor is attributed to the combined advantages of β-CD and GO. When compared with the commercial fibers and some published methods, the GO/β-CD sol-gel coating fiber showed a higher extraction efficiency, except for those organophosphate flame retardants containing a benzene ring. Similarly, Cao et al. combined the advantages of graphene and cyclodextrins with ionic liquids and ILs (Cao et al., 2019). They synthesized a VOIm^+^ ${\text{AQSO}}_{3}^{-}$ functionalized β-cyclodextrin/magnetic graphene oxide material (Fe_3_O_4_@SiO_2_/GO/β-CD/IL), which was used as a sorbent to extract plant growth regulators from vegetable samples using magnetic solid-phase extraction (MSPE) followed by UHPLC-MS/MS analysis. This approach showed fast separation, high surface area, high adsorption capability, and environmental friendliness. The comparison between Fe_3_O_4_@SiO_2_/GO, Fe_3_O_4_@SiO_2_/GO/β-CD, and Fe_3_O_4_@SiO_2_/GO/IL showed that Fe_3_O_4_@SiO_2_/GO/β-CD/IL had higher extraction efficiency and selective adsorption capacity.

For food analysis, β-CD combined with GO were used in the sample preparation during the analysis of organochlorine pesticide residues in honey (Mahpishanian and Sereshti, 2017). The prepared material was applied as a sorbent in vortex-assisted magnetic solid-phase extraction (MSPE) before gas chromatography-electron capture detection (GC-ECD) analysis. The method was optimized and evaluated, showing linearity ranging from 2 to 10,000 ng kg^−1^ and *R*^2^ > 0.9966, RSDs < 7.8%, LODs from 0.52–3.21 ng kg^−1^, and LOQ from 1.73–10.72 ng kg^−1^. For the real samples, the proposed sorbent showed good recoveries in the range of 78.8–116.2% with RSDs (*n* = 3) below 8.1%.

These works demonstrated that cyclodextrins' functionalized GBMs possess great supramolecular recognition, high extraction efficiency, good recoveries, and enrichment capability. It is noteworthy that in all reported works, the chosen graphene-based material is actually the graphene oxide (GO). This trend is justified by the presence of epoxy and -COOH groups on the GO surface, favoring the bonding with the CDs. Although some works reported the employment of graphene combined with cyclodextrins as sorbent, these materials were obtained by graphene oxide reduction (Ragavan and Rastogi, 2017; Tan and Hu, 2017). In this strategy, the reduction stage can be performed before or after the support between graphene-based and CDs. In this way, considering that the oxygenated groups present in the GO structure can improve interaction with molecules of β-CD (Tan and Hu, 2017), it is presumable that the reduction of graphene oxide after β-CD coupling is the best synthesis route to maximize the amount of cyclodextrin coupled.

To complement this topic, Table 2 presents recently published works using graphene-based materials combined with cyclodextrins for sample preparation. It must be highlighted that all applications employed β-cyclodextrin. Considering the existence of over 100 commercially available and more than 1,500 derivative materials already described, it is clear that sorbents based on graphene functionalized with cyclodextrins are a broad research field to be still explored. Finally, the GBMs/CD's excellent characteristics for supramolecular recognition, high extraction efficiencies, good recoveries, and enrichment capability should contribute to its widespread development in the coming years.

**Table 2:** Recent applications (2017–2020) of graphene-based materials functionalized by cyclodextrins to sample preparation.

**Sorbent**	**Analytes**	**Matrix**	**Sample preparation**	**Analysis**	**LOD**	**References**
G-Fe_3_O_4_-β-CD	Bisphenol-A	Water	MSPE	UV-vis	^**^	Ragavan and Rastogi, 2017
GNS/β-CD	Phenolphthalein	Water	d-SPE	UV-vis	^**^	Tan and Hu, 2017
Fe_4_O_3_-GO-β-CD	Neonicotinoid pesticide	Water	MSPE	HPLC-MS/MS	^**^	Liu G. et al., 2017
β-CD/MrGO	Organochlorine pesticides	Honey	MSPE	GC-ECD	0.52–3.21 ng kg^−1^	Mahpishanian and Sereshti, 2017
GO/β-CD sol-gel coating fiber	Organophosphate flame retardants	Environmental water	HS-SPME	GC-NPD	1.1–60.4 ng L^−1^	Deng et al., 2017
Fe_3_O_4_@SiO_2_/GO/β-CD/IL	Plant growth regulators	Vegetables	MSPE	UHPLC-MS/MS	0.01–0.18 μg kg^−1^	Cao et al., 2019

** *Not specified*.

### Magnetic Materials

Although graphene and its derivatives are considered to be cutting-edge materials in modern sorbent-based sample preparation (Toffoli et al., 2018; Grajek et al., 2019; Hou et al., 2019), their use can be related to some drawbacks in both bed-packed and dispersive microextraction. Their strong van der Waals interactions may cause irreversible aggregation of the material, causing graphene swelling, which often occurs due to the continuous water/solvent deposition between the graphene nanosheets (Zheng et al., 2017; Iakunkov et al., 2019). For these reasons, columns and microextraction devices packed with GBMs are usually susceptible to clogging and high backpressures. Likewise, for dispersive techniques, graphene nanosheets are well-suspended in solution, creating difficulty for the sorbent recovery, even after filtration and centrifugation (Hou et al., 2019; Li F. et al., 2020).

Within such a context, a modern, and advantageous strategy to overcome those drawbacks is the magnetic solid-phase extraction (MSPE) which is considered an efficient and environment-friendly sample preparation technique (Šafariková and Šafarik, 1999). MSPE extraction mechanism relies on the use of extraction sorbents supported over magnetic materials (Laura et al., 2019). In general, MSPE is a dispersive technique—thin-films or blocks format are also possible—in which the sorbent collection from the sample bulk is easily performed by application of an external magnetic field (Ibarra et al., 2015; Evrim et al., 2019). The use of graphene-based materials for MSPE not only efficiently eliminates the clogging problems from the packed-dispositive but can also enhance the extraction capacity due to GBMs' properties. Also, MSPE possibly eliminates additional centrifugation and filtration steps (Li et al., 2018). For these reasons, the use and development of magnetic sorbents incorporating GBMs have become a key-point in sample preparation in recent years. Nowadays, this combination has been applied in the MSPE of a wide diversity of organic and inorganic analytes from several complex samples, including the treatment of solid matrices (Feriduni, 2019).

These graphene-based magnetic sorbents are currently obtained by physical or chemical anchoring of the magnetic carries onto the graphene sheets. The most common carries include iron (Fe), cobalt, (Co), and nickel (Ni) oxides, highlighting the magnetite (Fe_3_O_4_) and maghemite (γ-Fe_2_O_3_) as the magnetic nanoparticles (MNPs) more frequently used (Laura et al., 2019). The Fe_3_O_4_ and γ-Fe_2_O_3_ have superparamagnetic properties, are easy to prepare, and disperse very well in aqueous solutions. Besides, those are MNPs feasible to be modified and functionalized (Yu M. et al., 2019). More popular methods for the preparation of Fe_3_O_4_ and γ-Fe2O3 based magnetic sorbents include chemical co-precipitation, hydrothermal synthesis, sol-gel reactions, solvothermal synthesis, thermal decomposition, microemulsion, and sonochemical approaches (Filik and Avan, 2019).

**Figure 5:**
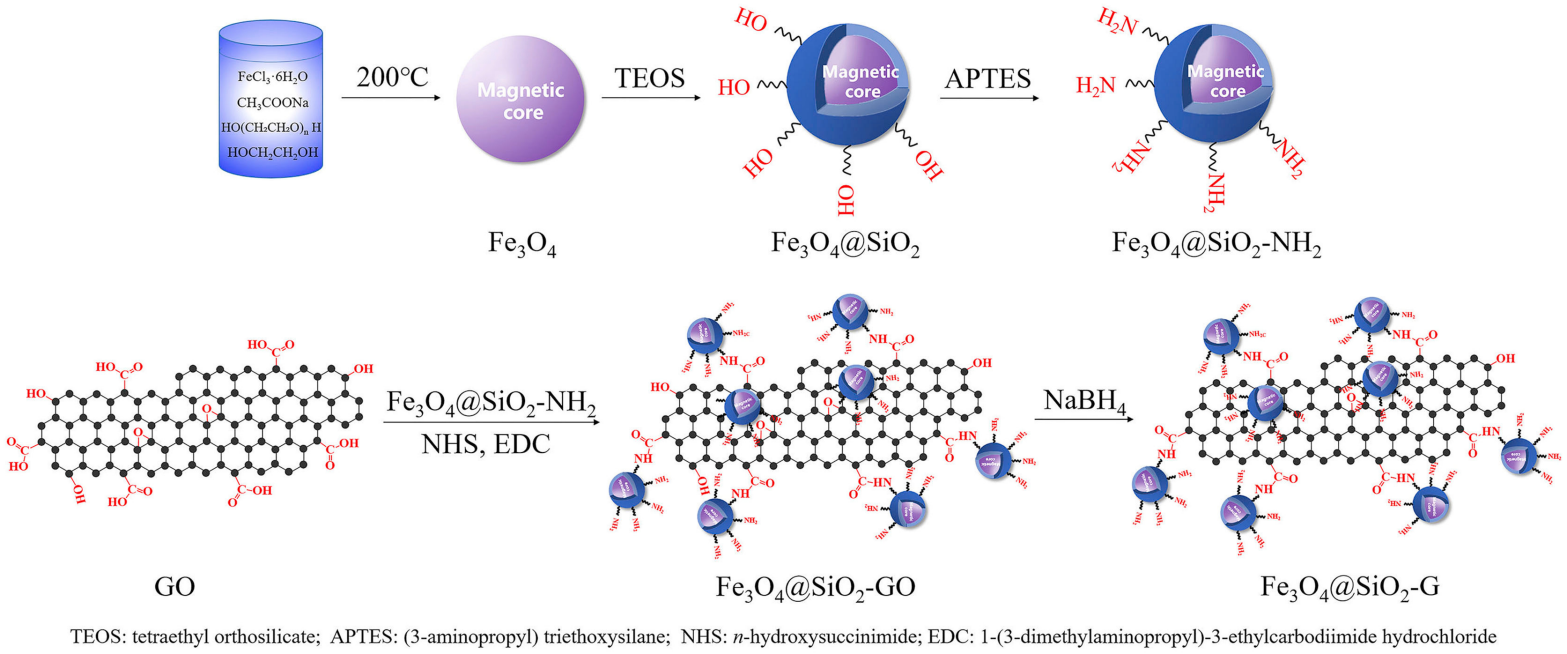
Schematic representation of GO loaded with Fe3O4 nanoparticles. Reprinted with permission from Yang et al. (2009), copyright 2009, Royal Society of Chemistry.

The most straightforward type of graphene magnetic sorbents is prepared by direct immobilization of the MNPs on the surface of the G. In this context, two synthetic routes can be employed for obtaining them: (i) the chemical co-precipitation and (ii) the hydrothermal synthesis. The chemical co-precipitation is based on the deposition of iron ions over the nanosheets by adding an alkaline solution to an aqueous dispersion of graphene Fe^3+^/Fe^2+^ salts, at elevated temperature and vigorous stirring. For example, this method was employed by Yang et al. to prepare superparamagnetic GO/Fe_3_O_4_ nanoparticles (Yang et al., 2009). After primary treatment of GO sheets, a dispersion of GO, FeCl_3_ was stirred under inert atmosphere by several hours. After that, Fe^3+^/Fe^2+^ ions were coordinated by the carboxylate anions on the GO sheets, and then GO/Fe_3_O_4_ nanoparticles were precipitated by the addition of an aqueous NaOH solution (Figure 5). As Fe^3+^ shows higher affinity than Fe^2+^ for carboxylic groups, the ratio of those ions should be controlled—generally, 2:1 is used—and the content of the carboxylic acid groups on the GO sheets needs to be previously determined by acid-base titration (Yang et al., 2009). The chemical co-precipitation method can also be employed to prepare magnetic reduced graphene oxide sorbents (rGO/Fe_3_O_4_). As an example, Chandra et al. prepared an rGO/Fe_3_O_4_ for arsenic removal from surface water samples (Chandra et al., 2010). In this case, after dispersion and precipitation with ammonia, GO/Fe_3_O_4_ particles were reduced to rGO/Fe_3_O_4_ by slowly adding hydrazine hydrate under stirring conditions 90°C.

An important issue is that the morphology of graphene magnetic sorbents prepared via chemical co-precipitation can be challenging to control. Thus, the resulting magnetic material sometimes presents low absorptivity due to the uneven distribution and agglomeration of the Fe_3_O_4_ particles on the nanosheets. Within such a context, hydrothermal synthesis has been proposed as an alternative to yield sorbents with better Fe_3_O_4_ particles distribution with more G exposed adsorption sites and then, improved adsorption capacity. This method is based on the reduction of Fe^3+^ and GO in sodium acetate and polyethyleneglycol in an autoclave at elevated temperature (Li et al., 2018). In this way, Wu and cookers prepared rGO/Fe_3_O_4_ particles (Wu et al., 2013), sonicating GO first in ethylene glycol, and then in the presence of FeCl_3._ After complete dispersion, the obtained clear solution was spiked with sodium acetate, and the mixture was sealed in a Teflon-lined stainless-steel autoclave and maintained at 200°C for 8 h. The authors reported regular morphology particles.

**Figure 6:**
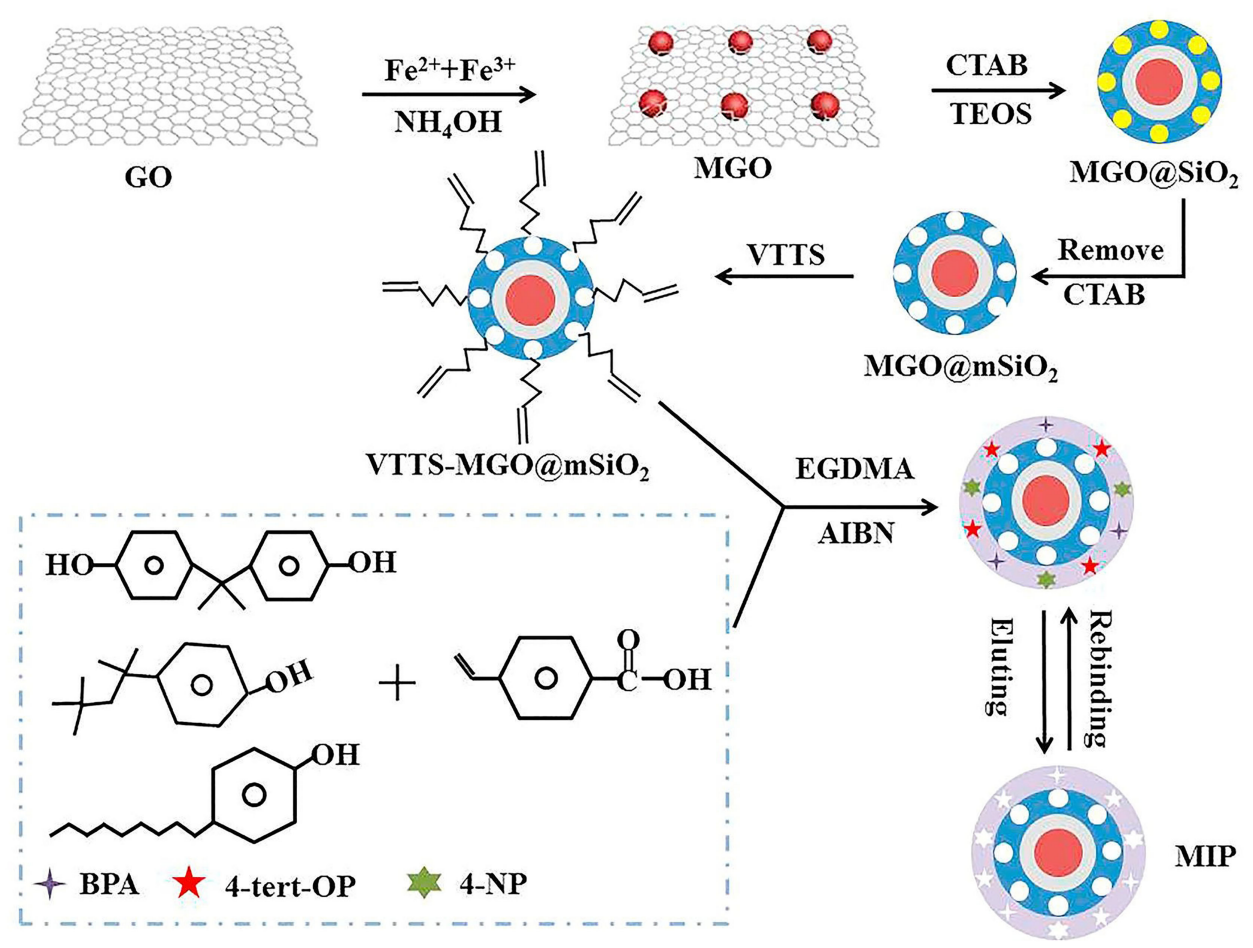
Schematic procedures for the preparation of Fe_3_O_4_@SiO_2_/G. Reprinted from Li et al. (2018) by permission from Elsevier, 2018.

Also, more reproducible, stable, and versatile graphene magnetic sorbents can be prepared from silica-coated magnetite particles (Fe_3_O_4_@SiO2). In this case, before graphene anchoring, Fe_3_O_4_ particles are modified with a silane coupling agent, consisting of tetraethyl orthosilicate (TEOS) and (3-aminopropyl) triethoxysilane (APTES) (Li et al., 2018). Sequentially, the graphene can be coupled to the particles by physical adsorption or by covalent bonding. Luo et al. prepared Fe_3_O_4_@SiO_2_/G particles for extraction of sulfonamides from water samples, by physical immobilization of graphene nanosheets on silica-coated magnetite (Luo et al., 2011b). The procedure obtained by pure dispersion under sonication for several hours renders particles not stable enough to continue reusing. Therefore, the chemical bonding of the graphene to the silanol groups is the preferred synthetic method. Amino groups are introduced on the surface of the Fe3O4@SiO2 particles and GO, anchored via an amidation reaction with the aid of cross-linking agents such as 1-(3-dimethyl aminopropyl)-3-ethyl carbodiimide hydrochloride (EDC) and -hydroxysuccinimide (NHS). This process is schematically represented in Figure 6 (Li et al., 2018). For example, Zhang et al. prepared Fe_3_O_4_@SiO_2_/GO particles by mixing Fe_3_O_4_@SiO_2_ and 3-aminopropyltriethoxysilane in isopropanol, under N_2_ atmosphere at 70°C, followed by addition of a GO solution containing NHS and EDC, stirring overnight (Zhang et al., 2014). Like Fe_3_O_4_/rGO composites, Fe_3_O_4_@SiO_2_/rGO particles can be obtained by the posterior reduction of GO with hydrazine.

The adsorption capacity of these magnetic particles is mostly based on the hydrophobic interactions of the honeycomb-like lattice into the carbon atoms. Consequently, bare graphene magnetic particles are not suitable MSPE sorbents for polar or ionic compounds. The main advantage of the Fe_3_O_4_@SiO_2_/G particles is the possibility to covalently bond additional functional moieties, which improve the adsorption capacity, selectivity, and applicability of them. In this manner, Fe_3_O_4_@SiO_2_/G have been covalently functionalized with ionic surfactants, ionic liquids (ILs), deep eutectic solvents, boronate affinity materials (BAM), supramolecules (crown ethers, cyclodextrins, calixarenes, cucurbiturils, and pillararenes), aptamers, polymers, and metal-organic frameworks (MOFs). For those magnetic GBMs, their preparation and applications were recently comprehensively reviewed by Li et al. They provided a summary of the state of the art of them, highlighting application as MSPE sorbents of organic compounds, biomolecules, and metal ions (Li et al., 2018). For this reason, herein, we provide an updated overview of the reported magnetic graphene sorbents between 2019 and 2020, and their applications in the determination of small organic molecules by chromatographic analysis.

In this context, magnetic graphene sorbents have found spread applications in the extraction of biomolecules organic compounds and metal ions. An assessment in the Scopus database, using the keywords “graphene” and “magnetic solid-phase extraction,” yield 248 results from 2010. Among them, 26 correspond to review papers and the rest to research papers mainly dedicated to describing the application of magnetic graphene sorbent in different areas of the analytical chemistry. As mentioned previously, Li et al. recently published a summary of the applications of graphene-based MSPE. In Table 3, we provide a summary of the magnetic graphene sorbent applications published from 2019 to date. It is noteworthy that a vast number of researchers using graphene-derived magnetic materials focus their efforts in areas as environmental surveillance and food security applications. This includes a wide diversity of analytes such as drug residues, pesticides, hormones, food additives, and active plant ingredients.

**Table 3:** Recent applications (2019–2020) of graphene-based MSPE to the determination of organic compound by chromatography and mass spectrometry.

**Sorbent**	**Analytes**	**Matrix**	**Analysis**	**LOD**	**References**
Magnetic N-doped 3D graphene-like framework carbon (Fe3O4@N-3DFC)	Cephalosporin antibiotics	River water and zebrafish samples	HPLC-UV	0.20–0.45 μg L^−1^	Niu et al., 2020
Fluorine and nitrogen functionalized magnetic graphene (G-NH-FBC/Fe2O3)	Perfluoroalkyl and polyfluoroalkyl substances	Water and functional beverage	HPLC-Orbitrap HRMS	3 ng L^−1^	Xian et al., 2020
Graphene-like MoS2-modified magnetic carbon-dot nanoflowers (MoS2@Fe3O4@C-dot NFs)	Ibuprofen	Pharmaceutical, environmental water and synthetic urine samples	HPLC-DAD	11 × 10^−6^ μg mL^−1^	Yilmaz and Sarp, 2020
Oxide/lanthanum phosphate nanocomposite (MGO@LaP)	Chlorpyrifos pesticides	Water and fruit samples	GC–ECD	0.67 μg L^−1^	Asadi et al., 2020
Fe3O4/rGO	Oral anticoagulants	Human plasma	HPLC-DAD	0.003 μg mL^−1^	Ferrone et al., 2020
Iron crosslinked alginate encapsulated magnetic graphene oxide (Fe/alginate/Fe3O4/rGO)	Endocrine-disrupting compounds	Superficial water	HPLC-UV	8–14 ng L-1	Shah and Jan, 2020
chitosan functionalized magnetic graphene oxide nanocomposite (Fe_3_O_4_@SiO_2_@CS/GO)	Alkaloids	Chinese herb (*Pericarpium papaveris*)	UHPLC-MS/MS	0.016–0.092 μg kg^−1^	Tang et al., 2020
Fe_3_O_4_/rGO	Oral anticoagulants	Human plasma	UHPLC-DAD	0.003 μg mL^−1^	Tang et al., 2020
Magnetic carbon nanodot/graphene oxide hybrid material (Fe_3_O_4_@C-nanodot@GO)	Ibuprofen	Human blood	HPLC-DAD	8.0 ng mL^−1^	Yuvali et al., 2020
Fe_3_O_4_/GO	Psychoactive drugs	Urine	UHPLC-MS/MS	0.02–0.2 μg L^−1^	Lu et al., 2020
Fe_3_O_4_/GO	Melamine	Water and dairy products	HPLC-UV	0.03 μg L^−1^	Abdolmohammad-zadeh, 2020
Three-dimensional graphene aerogel combined with Fe_3_O_4_ nanoparticles (3DG-Fe_3_O_4_@Sp)	Cholecalciferol (vitamin D3)	Bovine milk	HPLC-UV	3.01 μg L^−1^	Sereshti et al., 2020
Fe3O4/GO	45 multi-class pesticides	Vegetables (cabbage, leek, and radicchio)	GC-MS	0.4–4.0 μg kg−1	Chatzimitakos et al., 2019
Aptamer-functionalized Fe_3_O_4_/graphene oxide (Fe_3_O_4_/GO/Apt)	Chloramphenicol	Honey and Milk	HPLC-DAD	0.24 μg L^−1^	Tu et al., 2019
Flower-like hybrid material composed of Fe_3_O_4_, graphene oxide and CdSe nanodots (Fe_3_O_4_/GO/CdSe)	Ibuprofen	Pharmaceuticals, water, and urine	HPLC-DAD	0.36 ng mL^−1^	Sarp and Yilmaz, 2019
Fe_3_O_4_/rGO	Aflatoxin B1 and B2	Vegetable Oils	HPLC-PCD-FLD	0.01–0.02 μg kg^−1^	Yu L. et al., 2019
Fe_3_O_4_/rGO	N-nitrosamines	Mainstream cigarette smoke	HPLC-MS/MS	0.018–0.057 ng cigarette^−1^	Pang et al., 2019
GO@NH_2_@Fe_3_O_4_	Quinolones	Water	MALDI-TOF MS	0.010 mg L^−1^	Tang H. et al., 2019
GO@NH_2_@Fe_3_O_4_	Triazines	Water	DART-MS	1.6–152.1 ng L^−1^	Jing et al., 2019
Magnetic nitrogen-doped reduced graphene oxide (Fe_3_O_4_@N-rGO)	Endocrine disruptors	Carbonated beverages	HPLC-DAD	0.1–0.2 μg L^−1^	Li et al., 2019b
Molecular imprinted polymer (MIP) material combined with magnetic graphene oxide (Fe_3_O_4_/GO-MIP)	Quercetin and luteolin	Green tea and serum samples	HPLC-UV	0.09–4.5 ng mL^−1^	Dramou et al., 2019
Magnetic graphene-like molybdenum disulfide nanocomposite	Triazines and sulfonylurea herbicides	Water	UHPLC-MS	20 and 170 ng L^−1^	Zhou Y. et al., 2019
Ionic liquid magnetic graphene (IL@MG)	Microcystins	Water	UHPLC-MS/MS	0.414 ng L^−1^ and 0.216 ng L^−1^	Liu X. et al., 2019
Covalent organic framework-derived hydrophilic magnetic graphene Composite (magG@PDA@TbBd)	Phthalate esters	Milk	CapillaryLC-MS	0.004–0.02 ng mL^−1^	Lu et al., 2019a
Curcumin loaded magnetic graphene oxide	Parabens	Toothpaste and mouthwash	HPLC-DAD	0.4–1.0 ng mL^−1^	Razavi and Es, 2019
Fe_3_O_4_/rGO	Non-steroidal Anti-inflammatory Drugs	Animal food	HPLC-MS/MS	0.1–0.5 μg kg^−1^	Wang et al., 2019
Fe_3_O_4_/rGO	Phenolic compounds	Oil seeds	LC-MS/MS	0.02–90.00 μg kg^−1^	Lang et al., 2019
Fe_3_O_4_/rGO	Pesticides	Water	HPLC-UV	0.2–1.6 ng mL^−1^	Madej et al., 2019
Three-dimensional hierarchical frameworks based on molybdenum disulfide-graphene oxide-supported magnetic nanoparticles (Fe_3_O_4_/GO/MoS_2_)	Fluoroquinolone antibiotics	Water	HPLC-UV	0.25–0.50 ng mL^−1^	Xiao et al., 2019
Magnetic nanoparticles/graphene oxide (TPN/Fe_3_O_4_NPs/GO) nanocomposite	Pesticides	Water	HPLC-UV	0.17–1.7 μg L^−1^	Moradi et al., 2019
Silver-modified Fe_3_O_4_/graphene nanocomposite (Ag@Fe_3_O_4_@G)	Aromatic amines	Water	HPLC-UV	0.10–0.20 μg L^−1^	Alasl et al., 2019
Fe_3_O_4_/GO	Chlorophenols	Sewage water	GC-ECD	3.0–28.4 ng L^−1^	Esfandiarnejad and Sereshti, 2019
Ternary nano-composite, magnetite/reduced graphene oxide/silver (Fe_3_O_4_/rGO/Ag)	Morphine and codeine	Blood and urine	HPLC-UV-VIs	1.8–2.1 ng L^−1^	Abdolmohammad-zadeh et al., 2019
Magnetic amino-functionalized zinc metal-organic framework based on a magnetic graphene oxide composite (M-IRMOF/Fe_3_O_4_/GO)	Heterocyclic fungicides	Lettuce	HPLC-MS/MS	0.21–1.0 μg L^−1^	Liu G. et al., 2019
Fe_3_O_4_/rGO	Chiral pesticides	Cucumber, tomato, cabbage, grape, mulberry, apple, and pear	Chiral HPLC-MS/MS	0.02–10.0 μg g^−1^	Zhao et al., 2019
Reduced graphene oxide-carbon nanotubes composite (Fe_3_O_4_/rGO-CNTs)	Sulfonamides	Milk	HPLC-UV	0.35–1.32 μg L^−1^	Feng et al., 2019
Magnetic graphene oxide/multiwalled carbon nanotube core-shell (GO/MWCNT/Fe_3_O_4_/SiO_2_)	Paracetamol and caffeine	Synthetic urine and wastewater	HPLC-UV	0.48–3.32 ng mL^−1^	Ibrahim et al., 2019
Magnetized graphene oxide functionalized with hydrophilic phytic acid and titanium(IV) (magGO@PEI@PA@Ti^4+^)	Nucleobases, nucleosides, and nucleotides	Medicinal mushroom *C. Sinensis*, and natural foods	HPLC-DAD	1.8–2.8 ng mL ^−1^	Zhang Q. et al., 2019
MagG@SiO_2_@ZIF-8 composites	Phthalate Easers	Human Plasma	GC-MS	0.003–0.01 ng mL^−1^	Lu et al., 2019b
Multi-templates molecularly imprinted polymer (MIP) on the surface of mesoporous silica-coated magnetic graphene oxide (MGO@mSiO_2_),	Alkylphenols	Water	HPLC-DAD	0.010–0.013 μg L^−1^	Xie et al., 2019
GO	Phthalate esters	Bottled, injectable and tap waters	HPLC-UV	0.004–0.013 mg L^−1^	Abdelghani et al., 2019
Fe_3_O_4_/rGO	Non-steroidal anti-inflammatory drugs and bisphenol-A	Tap water	HPLC-DAD	0.031 mg L^−1^ and 0.023 mg L^−1^ 0.1785 mg L^−1^	Ungku Abdullah et al., 2019
rGO/ZnFe_2_O_4_ nanocomposite	Estrogens	Water, soil, and fish	UHPLC-QTOF-MS	0.01–0.02 ng mL^−1^	Li W. et al., 2020
Polydopamine functionalized magnetic graphene (PDA@MG)	Triazole fungicides	Water	HPLC-UV	4.8–8.4 ng L^−1^	Xiong et al., 2019
zeolitic imidazolate framework-7@graphene oxide (mag-ZIF-7@GO)	Fungicides	Water and soil samples	HPLC-Orbitrap HRMS	0.58–2.38 ng L^−1^	Zhang S. et al., 2019
Magnetic polyethyleneimine modified reduced graphene oxide (Fe_3_O_4_@PEI-rGO)	Polar non-steroidal anti-inflammatory drugs	Water	HPLC-DAD	0.2 μg L^−1^	Li et al., 2019a
Guanidinium ionic liquid modified magnetic chitosan/graphene oxide nanocomposites (GIL-MCGO)	DNA	Human whole blood and *E. coli* cell lysate	^**^	^**^	Liu M. et al., 2019

** *Not specified*.

### Molecularly-Imprinted Polymers

**Figure 7:**
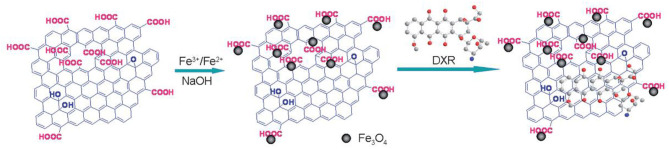
Schematic diagram representing the synthesis of VTTS-MGO@mSiO2@MIP. Reprinted with permission from Xie et al. (2019), copyright 2019, Elsevier.

Another group of materials explored for sample preparation involves the molecularly-imprinted polymers (MIPs) combined with GBMs. In this context, MIPs are prepared from a template (mold) which behaves structurally similarly as the target analytes, to achieve a selective interaction through a template-complementary binding site (Pan et al., 2018). These materials are traditionally prepared by copolymerization of the complex formed between the template and a functional monomer. This can occur through either covalent (hydrogen bonds) or non-covalent bonds (ionic and hydrophobic interactions) with a cross-linking agent in the presence of a suitable porogenic solvent (Zhou T. et al., 2019). After that, the molecular template is eliminated, resulting in a rigid three-dimensional cavity selective to the target analytes. As an example, Figure 7 depicts the synthesis steps employed by Xie et al. (2019). The authors selected 4-vinylbenzoic acid as a functional monomer to prepare the template taking advantage of the presence of the carboxyl ring and benzene, which can bind to BPA, 4- tert-OP, and 4-NP through hydrogen bonds and π-π interactions in the polymerization process.

Due to this high selectivity and relatively easy preparation, these materials have been widely used for molecular recognition and separation in different fields (sensors, drug delivery, protein recognition, and chromatography). In this section, their application in the field of sample preparation combined with GBMs is covered, highlighting preparation strategies of aqueous-recognition MIPs cleverly to achieve such functionalization (Zhou T. et al., 2019). For example, Luo et al. (2017) synthesized boronic acid-functionalized with graphene oxide, with a subsequent immobilization of ovalbumin as MIP-template, to obtain a high-selective sorbent, namely GO-APBA/MIPs. This strategy was chosen to overcome such difficulties related to specific recognition and separation of glycoproteins in complex biological samples. A comparison between the resulting material (GO-APBA/MIPs) and a bare GO-MIPs, without insertion of boronic acid, showed more extraction performance for the hybrid obtained sorbent. In this way, the outstanding recognition capacity by linking boronic acid and MIP cavities together with a high surface area of GO can represent a promising strategy to produce high performative sorbent material for biological glycoproteins.

Another interesting example was reported by Cheng et al. (2017), consisting of a more straightforward strategy using GO combined with MIP to extract and efficiently pre-concentrate bis(2-ethylhexyl) phthalate (DEHP) in environmental water samples. Contrary to the prior study (Luo et al., 2017), considering the smaller complexity of the target compound and water samples, the employment of only GO-MIP was already enough to achieve excellent extraction performance. In this case, dispersive solid-phase microextraction combined with HPLC-UV reported enrichment factors of more than 100-fold compared to the directly injected extract, highlighting this simple GO-MIP as a suitable sorbent in such cases.

An attractive approach to obtain high performative material involves the use of the graphene-based MIP functionalized with a magnetic particle to favor the extraction and sorbent isolation. Within such a context, Ning et al. (2014) proposed a novel nanosized substrate imprinted polymer (GO-MIP-Fe_3_O_4_) on magnetic graphene oxide (GO-Fe_3_O_4_) surface to remove 17β-estradiol (17β-E_2_) from food samples. The resulting sorbent has shown a functional extraction recovery of 84.20% at a low concentration level of 0.5 μmol L^−1^. Furthermore, due to the magnetic properties of the GO-MIP-Fe_3_O_4_, a simple, fast, and efficient separation of 17β-E_2_ were achieved, suggesting the combination between these materials as an excellent way to obtain hybrid sorbents. Following the same trend, Barati et al. (2017) synthesized a MIP based on magnetic-chitosan functionalize with GO to extract fluoxetine from environmental and biological samples. From our viewpoint, the outstanding characteristic of this work is the excellent pre-concentration factor of 500 related to such sorbent, which reinforces the combination of MIP, GBMs, and magnetic materials as an excellent way to improve sample preparation performance. Finally, another study based on a similar approach was presented by Fan et al., who prepared, through a chemical co-precipitation method, a novel hybrid sorbent based on MIP, GO, and superparamagnetic Fe_3_O_4_ particles (GO-MIP-Fe_3_O_4_). In this case, the author worked with natural samples, specifically alkaloids (evodiamine and rutaecarpine) extract from *Evodiae Fructus*, suggesting a great versatility of such magnetic GO-MIP sorbent. Also, its analytes' recovery achieves values over 82% considering as good values from our viewpoint.

Moreover, Table 4 presents other published studies to complement the discussion regarding hybrid sorbents combining MIP with GBMs.

**Table 4:** Applications of MIPs-GO composite in sample pretreatment.

**Sorbent**	**Analytes**	**Matrix**	**Sample preparation**	**Analysis**	**LOD**	**References**
GO-APBA/MIP	Ovalbumin	Egg white	SDS	Gel-Electrophoresis	^**^	Luo et al., 2017
bis(2-ethylhexyl) phthalate (DEHP)	Trace DEHP phthalate	Water	SPME	HPLC-UV	0.92 ng mL^−1^	Cheng et al., 2017
MIPs-GO-Fe_3_O_4_	17β-estradiol	Milk powder	External magnet	^**^	0.035 and 0.10 μmol L^−1^	Ning et al., 2014
GO-QDs-MIPs	p-t-octylphenol	Water	SPE	UHPLC-UV	0.15 μmol L^−1^	Han et al., 2015
(TFMAA)-GO (EGDMA)-GO	Cefadroxil	Water	d-SPE	UPLC-DAD	0.01 μg mL^−1^	Chen and Ye, 2017
MGR@MIPs	4-nitrophenol	Lake water	MIP	HPLC-UV	^**^	Luo et al., 2016
MIP-GO/Chm	Fluoxetine	Tap, well and spring water, and urine	MSPE	UV–Vis spectrophotometry	0.03 μg L^−1^	Barati et al., 2017
MIP@Fe_3_O_4_@GO	Evodiamine and rutaecarpine	*Evodiae fructus*	External magnet	HPLC-UV	^**^	Fan et al., 2017

** *Not specified*.

### Ionic Liquids

In 1914, a work with ionic liquids (ILs) was reported for the first time, although at that time, the author had no idea of the importance that these would take in the scientific area almost a century later (Walden, 2003). From the 1980's to the present, the interest in studying ionic liquids grew exponentially (Evans et al., 1981; Shi et al., 2015; Welton, 2018). Although they have been applied in several scientific areas, in the analytical chemistry the studies have focused mainly on the use of them for extraction, and separation purposes (Rodríguez-Sánchez et al., 2014; Tang et al., 2014; García-Alvarez-Coque et al., 2015; Marcinkowski et al., 2015; Hu et al., 2016; Yu et al., 2016; Nawała et al., 2018; Rykowska et al., 2018).

**Figure 8:**
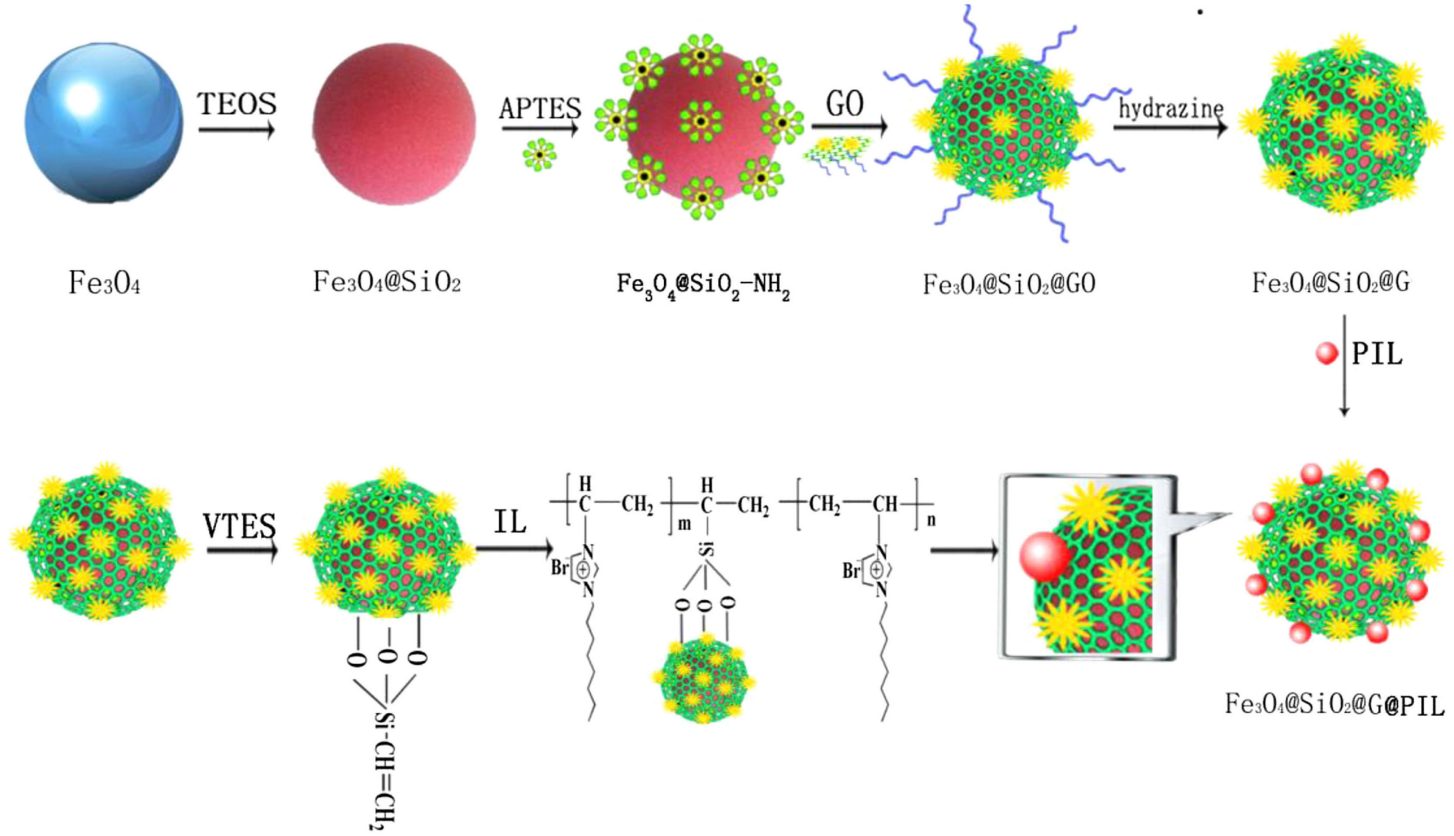
Preparation procedure of Fe_3_O_4_@SiO_2_@G@PIL. Reprinted with permission from Chen Y. et al. (2016), copyright 2016, Elsevier.

The ILs are compounds with a dual nature acting as nonpolar for nonpolar analytes and, inversely, for those with a strong proton donor group, depending on the separation mechanism that it presents (Berthod et al., 2018). Therefore, their excellent properties (high thermal stability, good solubility, and easily functionalization) have been modified in several different ways. One approach consists of replacing the anionic or cationic part with another material, of automatically regulating the IL property or nature (hydrophobicity, hydrophilicity, viscosity, and among others) (Ho et al., 2014; An et al., 2017). In this way, improvements in their sensitivity and selectivity to the extraction of the different analytes can be achieved. One of these desired enhancements is obtained by combining the ILs with GBMs, owing to their different types of chemical interactions with the analytes (e.g., n-π, π-π, hydrogen bonding, dipolar, ionic charge/charge) (Chen Y. et al., 2016; Feng et al., 2020). For these reasons, IL-GBMs present excellent extraction efficiency for a wide variety of analytes in several complex matrices (e.g., environmental, food, drinks, biological, and among others), as presented. Additionally, Figure 8 illustrates a typical synthesis process performed to achieve such hybrid sorbents.

The employment of ILs combined with GMBs includes the work reported by Liu X. et al. (2019) to determine the environmentally-dangerous monocyclic heptapeptides (microcystins, MC) in natural water samples. They synthesized the IL-G by the co-precipitation route. For the analytes' extraction, the MSPE technique was used while for its separation, determination, and quantification, the authors employed UHPLC-MS/MS. For the optimization of the experimental parameters, univariate analysis, and orthogonal screening were used. The analysis time was 18 min, with excellent linearity. The LODs were 0.414 and 0.216 ng L^−1^ for MC-LR and MC-RR, respectively, reporting traces of these two compounds in natural water samples, and it can be concluded that the method used is promising for the study of other types of microcystins in water samples.

Other interesting applications based on IL-GBMs include work published by Chen Y. et al. (2016). They synthesized magnetite nanoparticles (Fe_3_O_4_) of controlled size by a co-precipitation method to obtain the Fe_3_O_4_-SiO_2_-G-PIL hybrid sorbent; the obtained material was used to determine preservatives in vegetables by QuEChERS following GC-MS analysis. In this study, it was possible to take advantage of the functional properties related to magnetic nanoparticles and also by its coupling to the GO with the polymeric ILs (e.g., high surface area, and solvent effects). Moreover, the method's detection limits varied between 0.82 and 6.64 μg kg^−1^ for the 20 preservatives studied. Similarly, Tashakkori et al. (2019) synthesized a series of ionic liquids grafted onto stainless steel wires, which were previously coated with GO, using a sol-gel technique. The authors used this hybrid material as a sorbent in an on-line DI-SPME-GC-MS approach to determine phthalate esters (PAE) in several samples such as tap water and seawater and coffee. They reported low detection limits (5–30 ng L^−1^) and the lab-made SPME fibers being used more than 120 times, which is a useful feature when compared with the commercially available ones such as PA and CAR/PDMS. These results reinforce the great interest in developing hybrid GBMs combined with ILS.

Additionally, biological samples were analyzed by Ding et al. (2015). They functionalized magnetic chitosan with a series of ionic liquids of guanidinium and graphene oxide, aiming to extract trypsin, lysozyme, ovalbumin, and albumins from bovine serum. In this case, the analytical results obtained by this hybrid guanidinium-IL functionalized with MCGO were compared with those achieved by employing just GO, magnetic chitosan, or MCGO. In this case, the hybrid guanidinium-IL-MCGO exhibits higher extraction performance compared to the other sorbents, which suggests the combination of GBMs and ILs as a suitable strategy to achieve more performative extractive phases. Also, Hou et al. (2016) proposed a poly(1-vinyl-3-hexylimidazolium bromide)-GO-grafted silica [poly(VHIm^+^Br^−^)-GO-Sil] as a hybrid IL sorbent to extract flavonoids in urine samples. They synthesized the material by an interesting process consisting of an *in situ* radical chain-transfer polymerization and then *in situ* anion exchange. In this case, the procedure started with the silica coating by GO, using a manufacturing layer by layer fabrication method. Then, the poly (VHIm^+^Br^−^)-GO-Sil anion was transformed into hexafluorophosphate (PF^6−^) by *in situ* anion exchange. The method based on SPE-HPLC-UV showed acceptable extraction recoveries for four flavonoids, with limits of detection in the range of 0.1–0.5 μg L^−1^. The proposed material showed ecological and cost-effective advantages. It can be applied successfully to the extraction and enrichment of flavonoids in human samples, even allowing the study of metabolic kinetics.

Finally, considering other types of samples, an interesting study published by Zhou et al. (2016) was carried out to analyze phthalates (PAE) in eraser samples. The Office of Quality and Technology Supervision of the province of Jiangsu in China required PAE analyses in several samples to maintain concentrations inside the accepted limits registered by law. For this reason, it is necessary to monitor residues of this compound not only in food but also in everyday objects used by people (Zhou et al., 2016). Within such a context, a new graphene oxide compound modified with ionic liquids (GO-[AEMIM][Br]) was synthesized through an amidation reaction between the amino groups of the ILs and the carboxyl groups of GO. The high extraction capacity reported by this hybrid sorbent suggests the combination of the properties of ILs and GBMs (tunability and high surface area) as a positive relationship that assisted the PAE extraction in such a non-common sample.

Apart from the interesting application herein discussed, other relevant studies based on hybrid sorbents combining IL and GBMs are shown in Table 5.

**Table 5:** Applications of Ils-GO composite in sample pretreatment.

**Sorbent**	**Analyte**	**Matrix**	**Sample preparation**	**Analysis**	**LOD**	**References**
IL-GO@Silica	Chlorophenols	Water	SPE	HPLC-UV	^**^	Wang et al., 2017a
IL@MG	Microcystins	Water	MSPE	UHPLC-MS/MS	0.414 ng L^−1^ and 0.216 ng L^−1^	Liu X. et al., 2019
[C_4_C_12_im]@GO	Hg	Water	SPE	AAS	14 ng L^−1^	Sotolongo et al., 2018
[BMim]@MGO	Heavy metal ions	Water	ICP-OES	ICP-OES	0.1–1.0 μg L^−1^	Rofouei et al., 2017
MGONPs-C_16_mimBr	Chlorophenols	Water	MSPE	HPLC-UV	0.10–0.13 μg L^−1^	Liu W. et al., 2017
PGO-MILN	Chlorophenols	Water	MSPE	LC-MS/MS	0.2–2.6 ng L^−1^	Cai et al., 2016
GO-1-butyl-3-aminopropyl imidazolium chloride.	Anabolic steroids β-blockers	Water	SPE	HPLC-DAD	7–23 ng L^−1^	Serrano et al., 2016
GO-PILs monolith	Phenolic	Water	SPME	HPLC-UV	0.2–0.5 μg L^−1^	Sun et al., 2016
Fe_3_O_4_@SiO_2_@G@PIL	Preservatives	Vegetables	QuEChERS	GC-MS	0.82–6.64 μg kg^−1^	Chen Y. et al., 2016
rGO/ILN-ETD	Estrogens	Milk	ETD	HPLC-DAD	0.09–0.30 μg L^−1^	Chu et al., 2019
1-(3-aminopropyl)-3-vinyl imidazolium bromide/tetrafluoroborate) grafted	Phthalate Esters	Coffee Tap Water Seawater	SPME	GC-MS	5–30 ng L^−1^	Tashakkori et al., 2019
PANI-MWCNTs-rGO-IL	Alcohols	Tea	SPME	GC-FID	2.2–28.3 ng L^−1^	Li et al., 2016
IL-TGO	Auxins	Soybean sprouts	PT-SPE	HPLC-DAD	0.004–0.026 μg g^−1^	Zhang H. et al., 2018
MGO-C_16_MIM-DMG	Trace nickel	Spinach Cacao powder Tea Cigarette	MSPE	FAAS	0.16 μg L^−1^	Aliyari et al., 2016
MCGO@guanidinium IL	Protein	Bovine serum	MSPE	UV-vis	^**^	Ding et al., 2015
poly(VHIm^+^Br^−^)@GO@Sil	Flavonoids	Urine	SPE	HPLC-UV	0.1–0.5 μg L^−1^	Hou et al., 2016
IL-coated Fe_3_O_4_/GO	Hemin	Serum	SPE	FAAS	3.0 μg L^−1^	Farzin et al., 2016
1-(3-aminopropyl)imidazole chloride modified MGO	Polysaccharides	Brown alga	MSPE	HPLC-UV	^**^	Wang et al., 2017b
PILs@GO@Sil	Phenolic acids	Black wolfberry yogurt and urine	SPE	HPLC-UV	0.20–0.50 μg L^−1^	Hou et al., 2018a
Magnetic GO/PPy	Methotrexate	Urine	d-SPE	HPLC-UV	7 ng mL^−1^	Hamidi et al., 2019
3D-IL-Fe_3_O_4_-GO	PAHs	Human blood	PT-SPE	GC-MS	0.002–0.004 μg L^−1^	Zhang Y. et al., 2018
PIL(Br)-G/SiO_2_	Human serum albumin	Human whole blood	SPE	HPLC-UV	^**^	Liu et al., 2018
Fe_3_O_4_/GO NPs	Cephalosporins	Urine	MSPE	HPLC-UV	0.6 and 1.9 ng mL^−1^	Wu et al., 2016
GO-[AEMIM][Br]	Phthalates	Eraser	SPE	HPLC-UV	0.02–0.88 ng mL^−1^	Zhou et al., 2016

** *Not specified*.

### Miscellaneous

This section aims to present some alternative graphene-based materials that could be an alternative for the sample preparation in the future. For this reason, there are not many publications apart from the materials already discussed in the previous sections. An original example recently published by Tahmasebi (2018) consisted of intercalating aluminum polyoxocations (Al_30_) between the graphene oxide (GO) nanosheets and further support this onto polycaprolactone nanofibers, aiming to enhance temperature, and pH resistance to the sorbent phase. This novel material was applied in SPE extraction of four statin drugs showing acceptable analytical performance. The author emphasized the excellent ability of this sorbent to interact with acidic polar species, possibly due to the resistance to pH variations. Another interesting application was performed by Li et al. (2014) whose synthesized a magnetic ionic liquid/chitosan/GO (MCGO-IL) as a biodegradable/bio-sorbent for the mitigation of Cr (IV) in the treatment of residual in environmental water samples. A solid-liquid separation was performed in the presence of an external magnetic field, involving a pseudo-second-order kinetic sorption step. The maximum adsorption capacity was 145.35 mg g^−1^, obtained thanks to the intermolecular hydrogen bond between MCGO-IL and Cr(IV), due to the hydroxy and amine groups to which the ions bind metallic. This result demonstrates the potential of this hybrid sorbent material in the cleaning processes of contaminating metal ions in complex matrices such as water. Additionally, the authors emphasize its low producing cost as a useful characteristic since commercially available sorbents often are expensive and non-reusable.

Additionally, the more significant interlayer distance between GO sheets, due to Al_30_ insertions, can enhance the interaction with the π-electron system onto the GO surface, resulting in enhanced extraction performance. Similarly, Amiri et al. (2019) synthesized another hybrid sorbent but this time supporting GO nanosheets with polyoxotungstate (POT), instead of Al, to enhance chemical stability and pH resistance once POT act as charge-compensating and space-filling compound. This strategy is proposed to enhance the POT water dispersibility and surface area of the sorbent, possibly favoring the extraction performance while ensuring its high thermal and pH stability. Another strategy, underscored by Farajvand et al. (2018), was to covalently-bond an electrically conducting polymers onto the GO surface to diminish its self-aggregation in aqueous as well as enhance adsorption capacity. In this case, polyaniline was used considering its various oxidation states possessing distinct charge carriers which allow chemical interactions with several heavy metals. In this case, this sorbent was tested for Cd isolation from environmental water samples by SPE and subsequent dispersive liquid-liquid extraction.

In general, all these modifications performed in the G or GO chemical structures aim to improve its performance. However, a work published by Ashori et al. seems to follow the reverse trend focusing on using the graphene oxide as a reinforcement for glass fiber and epoxy composites. For this reason, the primary goals were to improve chemical reactivity, toughness, and adhesion to polymeric matrices, including the GO's widely-known properties. Although this material had not been applied for sample preparation yet, its high mechanical strength might be a promising tool particularly for miniaturized sample preparation techniques once they often demand high-pressure procedures causing clogging problems when GO or G are applied as sorbents. Recently, another interesting compound, namely graphene-aerogel, has gained attention mainly due to its superior and tunable volume as well as surface area as compared to graphene. The aerogel only itself exhibits poor extraction performance for water-soluble analytes, thus demanding some modification on it, as the functionalization of graphene-based compounds. In this way, graphene-based aerogels are suitable for sample preparation since they can relate the great qualities of graphene with the impressive compressibility of aerogels. Therefore, high-performance sorbents packed into sample preparation hardware can be reusable many times, considering the compressibility factor, which can help unpack and recover these extractive phases. As examples, Maggira et al. (2019) and Tang S. et al. (2019) reported two self-recoverable graphene-aerogels which were successfully applied to the analysis of sulfonamides and phenolic compounds in complex matrices, respectively.

Considering the impressive arising of graphene-based sorbents throughout the last years, herein, we aimed to pinpoint some of the unusual approached to perform such modifications and production of extractive phase. However, novel sorbent phases can be expected to be developed daily, considering the great qualities of nanomaterials for analytical chemistry purposes, highlighting the graphene.

## Concluding Remarks

Bearing in mind the great importance of sample preparation to clean-up samples, extract, and pre-concentrate target analytes become easier to understand the increasing interest of the analytical chemistry in developing modern strategies to optimize such a critical stage. In this context, one of the most relevant and promising fields is the development of more performative and environmentally-friend sorption-based techniques and, by consequence, the sorbents commonly used on them. As it is known, a good sorbent material must have some essential characteristics, including (i) selectivity for specific analytes and thus present chemical inertia for matrix interferents; (ii) good recovery and enrichment factors; and (iii) simple and non-expensive production. Once fulfilling these requirements, graphene-based materials have increasingly seemed to be the right candidate since their first application in sample preparation around 2011. Its large surface area, together with the π-π delocalized electron system, aid in improving so much the extraction performance of target compounds possessing aromatic rings as several chemical classes (e.g., pesticides, pharmaceuticals, and others) as herein discussed on section Graphene and Graphene Oxide.

Although the successful application in its “bare” form (G and GO), sometimes the functionalization of them seems to enhance even more the performance of such sorbents. For this reason, in this work, we have shown several different applications based on the use of hybrid materials consisting of GBMs anchored to other extractive sorbents such as alkyl and aryl groups, cyclodextrins, magnetic particles, molecularly imprinted polymers, ionic liquids, and among others. The functionalization is often achieved by forming covalent or hydrogen bondings, using sol-gel or polymerization router, or even electrochemical deposition. This interesting approach is encouraged by the possibility to ally the advantages of each class in only one. Apart from this goal, the clogging and backpressure problems often related to G and GO when packed in sorbent-based sample preparation techniques are overcome by the addition of other compounds. For example, several works underscored in sections Alkyl and Aril Groups, Cyclodextrins, and Magnetic Materials based on magnetic particles, or anchoring *in silica* reported this result.

Similarly, increasing on extraction selectivity and by consequence, performance is observed when GBMs were functionalized with both MIPS or the tunable-ILs, as can be seen in sections Molecularly-Imprinted Polymers and Ionic Liquids. This background explains the significant tendency to work with hybrid graphene-based materials instead of its bare form. GBMs are an excellent sorbent, often surpassing the commercially available phases such as C8 and C18.

After all, by assessing the recent literature and considering the vast number of applications involving graphene in the sample preparation arena, as herein discussed, an increasing tendency to expand the footprint of GBMs functionalized with several classes must continue in the years to come. This conclusion is mainly supported by the unique favorable GBMs physical-chemical properties, which—together with the advancements on the materials synthesis routes, extraction techniques, and related subjects—evidenced this field as one of the most critical developments in the sample preparation area nowadays. Also, an increasing number of papers reporting the employment of hybrid GBMs and miniaturized sample preparation techniques must be expected. This trend can be projected considering the high potential obtained by combining the well-established benefits of automation/miniaturization with the use of more selective and performative materials, possibly leading to greener sample preparation techniques by following the Green Chemistry concept.

## Author Contributions

EM wrote sections Introduction, Miscellaneous, and Concluding Remarks, as well as edited the whole manuscript. KM-C wrote sections Graphene and Graphene Oxide and Alkyl and Aril Groups, built Table 1, and revised the whole manuscript. MJ-S wrote sections Molecularly-Imprinted polymers and Ionic Liquids, as well as built Tables 4, 5. LS wrote section Cyclodextrins and built Table 2. DV wrote section Magnetic Materials and built Table 3. FL conceptualized, supervised, and edited all versions of the manuscript, as well as provide all required facilities. All authors contributed to the article and approved the submitted version.

## Conflict of Interest

The authors declare that the research was conducted in the absence of any commercial or financial relationships that could be construed as a potential conflict of interest.
